# Identification of key genes associated with muscle atrophy after spinal cord injury and experimental verification in rats

**DOI:** 10.3389/fimmu.2025.1673367

**Published:** 2026-01-27

**Authors:** Xiang Wang, Yimin Gao, Jianzhong Huo

**Affiliations:** 1 Department of Pain Medicine, Third Hospital of Shanxi Medical University, Shanxi Bethune Hospital,Shanxi Academy of Medical Sciences, Taiyuan, China; 2 Department of Gynaecology and Obstetrics, Third Hospital of Shanxi Medical University, Shanxi Bethune Hospital, Shanxi Academy of Medical Sciences, Taiyuan, China; 3 Department of Orthopedics, Second Hospital of Shanxi Medical University, Taiyuan, China

**Keywords:** differentially expressed genes, spinal cord injury, muscle atrophy, hub genes, bioinformatics analysis, weighted gene co-expression network analysis

## Abstract

**Background:**

Spinal cord injury (SCI) is a neurological disease with high morbidity and mortality. Post-SCI muscle atrophy is a cascade response to SCI, and failure to actively prevent its occurrence severely affects patients’ mobility and quality of life. Therefore, deeply exploring the correlation between muscle atrophy after SCI and the molecular regulation mechanism is of great significance.

**Methods:**

Download GSE21497 expression profile data from the gene expression omnibus (GEO) database. Perform weighted gene co-expression network analysis (WGCNA) on the obtained differentially expressed genes (DEGs). Subsequently, we performed functional and pathway enrichment analyses of key modules. Construct a protein-protein interaction (PPI) network and screen core genes. Finally, the results were verified by real-time polymerase chain reaction(PCR).

**Results:**

A total of 1007 DEGs were obtained, including 533 upregulated genes and 474 downregulated genes. WGCNA analysis identified 161 turquoise modules of DEGs as key modules related to SCI. Functional enrichment analysis showed that these genes were mainly enriched in negative regulation of cellular process, cytosol, response to organic substance, endpoint system, extracellar region, peroxisome proliferators-activated receptors (PPARs) signaling, adherens junction signaling, and DNA replication signaling pathway.

**Conclusions:**

FOS and CCL2 may be involved in the molecular pathophysiology of muscle atrophy after SCI, serving as potential targets for diagnosis or treatment of SCI-related muscle atrophy.

## 1 Introduction

Spinal cord injury (SCI) refers to a neurological disorder characterized by damage to the spinal cord, resulting from direct or indirect trauma, leading to dysfunction of motor, sensory, and autonomic functions below the level of injury within the neuroanatomical distribution (1). According to statistics, approximately 100,000 to 800,000 individuals in China experience SCI annually, while in the United States, each SCI patient incurs lifetime costs ranging from $1.1 million to $4.7 million, imposing significant burdens on both families and society, severely affecting the physiological, psychological health, and quality of life of patients (2, 3). With the rapid development of modern transportation, construction, and industry, the incidence and disability rate of SCI have been steadily increasing each year (4). Mechanical compression following SCI can cause direct mechanical damage to spinal cord tissue, including local hemorrhage, edema, and ischemic reactions, a process referred to as primary injury (5). This type of injury triggers a cascade of cellular and molecular responses, including inflammation, lipid peroxidation, free radical generation, disruption of ion channels, axonal demyelination, glial scar formation, necrosis, and programmed cell death, collectively termed secondary injury (6).

One of the most apparent consequences following SCI is a significant decrease in muscle mass due to partial or complete interruption of descending motor pathways (7). Post-SCI muscle atrophy is a cascade response to SCI, and failure to actively prevent its occurrence severely affects patients’ mobility and quality of life (8). Following SCI, changes occur in spinal cord motor neurons and nerve fibers, leading to dysfunction of motor units, which results in decreased muscle fatigue resistance, reduced strength, and slowed motor conduction velocity. These alterations exacerbate functional decline post-SCI and impede motor recovery (9). Studies have shown that after SCI, extracellular vesicles released by inflammatory cells contain pro-inflammatory factors. When these extracellular vesicles act on skeletal muscle cells, they activate intracellular inflammatory signaling pathways, leading to increased expression and release of inflammatory factors such as tumor necrosis factor-α (TNF-α) and interleukin-1β (IL-1β). This triggers an inflammatory response in skeletal muscle, accelerates muscle protein degradation, reduces protein synthesis, and ultimately promotes skeletal muscle atrophy (10). Celecoxib can inhibit the activity of cyclooxygenase-2 (COX-2), thereby reducing the expression of prostaglandin E2 (PGE2). It effectively blocks the activation of the NF-κB signaling pathway, inhibits the release of inflammatory cytokines such as TNF-α and IL-1β, and alleviates inflammatory damage to muscle tissue (11). Recent studies have also confirmed that tail nerve electrical stimulation can activate relevant neural circuits, prompting lumbar spinal cord neurons to synthesize neurotrophin-3 (NT-3). This increases the survival number of motor neurons in the lumbar anterior horn and maintains the synthesis of their cholinergic neurotransmitters, thereby effectively preventing or alleviating muscle atrophy in paralyzed hindlimbs (12). Given the potential modifiability of post-SCI muscle atrophy (13), studying its mechanisms and providing effective treatment strategies have become recent focal points. With the development of bioinformatics and the widespread use of high-throughput gene chip technology in exploring molecular changes in disease progression, there is now a theoretical basis and research direction for understanding the pathogenesis of post-SCI muscle atrophy (14). This facilitates the targeted selection of genes with research value, potentially saving significant research time and effort.

This study used the post SCI muscle atrophy expression profile data (GSE21497) from the gene expression omnibus database (GEO) as the research object, screened differentially expressed genes (DEGs), and performed weighted gene co-expression network analysis (WGCNA) and functional enrichment analysis to construct a protein protein interaction (PPI) network and screen for key genes. These analyses aim to provide a theoretical basis for exploring the molecular mechanisms underlying the development and maintenance of muscle atrophy post-SCI, as well as identifying important therapeutic targets for treating muscle atrophy. Finally, the results were verified by real-time polymerase chain reaction (PCR).

## 2 Materials and methods

### 2.1 Microarray data analysis

Microarray and gene expression profile dataset GSE21497 were obtained from the freely accessible public database NCBI-GEO (https://www.ncbi.nlm.nih.gov/geo/). The gene sequencing platform used was GPL570 [HG-U133_Plus_2] Affymetrix Human Genome U133 Plus 2.0 Array. The GSE21497 dataset includes 9 male and 1 female patients with SCI, with a mean age ± standard error of 43.9 ± 6.7 years. Among them, 6 patients suffered from tetraplegia and 4 patients from paraplegia. On the 2nd and 5th days after SCI, muscle biopsy samples were obtained from the vastus lateralis muscle of the patients. The online tool GEO2R (https://www.ncbi.nlm.nih.gov/geo/geo2r/) was utilized to perform DEGs analysis between the two days and five days post-SCI group.

### 2.2 Data processing and selection of DEGs

Using the online Sangerbox 3.0 (http://vip.sangerbox.com/home.html), we performed ID conversion and gene annotation processing on dataset GSE21497 (15). Differential analysis was conducted using the R software package limma (version 3.40.6) to obtain DEGs between the two groups (16). Based on the acquired expression profile dataset, the data were log2-transformed, and multivariate linear regression was performed using the lmFit function. Subsequently, the eBays function was employed to obtain the expression matrix, resulting in the identification of significant differences for each gene. DEGs were selected using a threshold of *P<*0.05 and |logFC|*>*1.5. The online Sangerbox 3.0 software was then utilized to input the original data in TXT format, facilitating online construction of DEGs and the generation of volcano plots and heatmaps.

### 2.3 Weighted gene co-expression network analysis

Performing weighted gene co-expression network analysis (WGCNA) using the R software package on the obtained DEGs (17).Inputting the DEGs data for sample selection, wherein sample and grouping information were used to set a cut-Height threshold for sample exclusion, removing outliers, and constructing sample clustering. After preparing the data for sample clustering observations, determining the optimal soft threshold (β) to ensure the constructed network conforms to a scale-free topology. Constructing a co-expression network based on the optimal soft threshold and partitioning genes into different modules, represented by branch colors on the clustering tree, and plotting gene clustering trees and module eigengene clustering diagrams. Based on these results, calculating the correlation between gene modules and phenotypes, identifying trait-associated modules, and plotting phenotype-gene correlation heatmaps.

### 2.4 Functional enrichment analysis

Gene Ontology (GO) and Kyoto Encyclopedia of Genes Genomes (KEGG) pathway enrichment analyses were conducted on DEGs utilizing the clusterprofiler in R package (version 3.14.3, http://bioconductor.org) (18, 19). Using the Database for Annotation, Visualization, and Integrated Discovery (DAVID, https://david.ncifcrf.gov/home.jsp) for online gene function annotation and signal pathway enrichment analysis (20), including biological processes (BP), molecular functions (MF), and cellular components (CC), as well as KEGG pathway analysis. Genes count *≥*2 and *P<*0.05 was used as the threshold of significant enrichment results.

### 2.5 Gene Set enrichment analysis

To observe whether there are changes in the distribution of pathway genes in all gene expression data between different time point groups of SCI patients. We obtained GSEA version3.0 from the GSEA website (http://software.broadinstitute.org/gsea/index.jsp) and divided the samples into two groups based on different observation time points (21). Subsequently, we downloaded the subset from the Molecular Signatures Database (http://www.gsea-msigdb.org/gsea/downloads.jsp) to assess relevant pathways and molecular mechanisms (22). Based on gene expression profiles and phenotype grouping, a minimum gene set of 5 and a maximum gene set of 5000 were established, with 1000 permutations. A significance threshold of *P<*0.05 and *FDR<*0.25 was considered statistically significant.

### 2.6 Construction of protein-protein interaction networks and identification of hub genes

Using the STRING online database (https://string-db.org/), the selected DEGs were inputted to analyze and construct PPI networks. The obtained data were imported into Cytoscape software to build protein co-expression network diagrams for comprehensive bioinformatics analysis (23). Using the CytoHubba plugin in Cytoscape, the top 30 genes were selected, and subsequently, the top 10 hub genes were determined using various algorithms including Degree, maximum clique centrality (MCC), maximum neighborhood component (MNC), and edge percolated component (EPC). CytoHubba comprehensively uses four algorithms, namely Degree, MCC, MNC, and EPC, to evaluate the global connectivity, local compactness, topological importance of nodes, and edge permeability respectively. Selecting multiple algorithms can avoid the bias of a single algorithm, and cross-validation with multiple algorithms can improve the robustness of the results. To ensure the reliability of the screening results, we adopt a two-step weighting strategy: algorithm weight assignment and comprehensive score calculation. The raw data of the identified hub genes were inputted into the online Sangerbox 3.0 software to find intersections between sets and to generate Venn diagrams. Organize and analyze the differential expression of hub genes at different stages. Conduct statistical analysis on the expression differences of hub genes in the presence of injuries of varying degrees at the same stage.

### 2.7 Establishment of a spinal cord injury model

Forty healthy, adult, female Sprague-Dawley (SD) rats of specific pathogen-free (SPF) grade were obtained from the Animal Experiment Center of Shanxi Medical University [Animal Use License Number: SYXK (Jin) 2019 0008], with body weights ranging from 210 to 250 g. The experimental procedures complied with ethical standards for animal care and use. This study was approved by the Animal Ethics Committee of Shanxi Bethune Hospital (Approval Number: SBQKL-2021-029). All methods were performed in accordance with ARRIVE guidelines and relevant and regulations. The rats were randomly assigned into two groups (n=20) using a random number table: the sham operation group (Sham group) and the spinal cord injury group (SCI group). In the Sham group, the rats underwent laminectomy and removal of the spinous processes at the T_8–9_ vertebrae without spinal cord injury. In the SCI group, spinal cord injury was induced using the Allen method (24): after excising the T_8–9_ spinous processes and laminae for complete exposure of the spinal cord, a weight of 5 grams of material drops from 10 cm height on the exposed spinal cord, causing damage. The success of the model was determined when the rats displayed tail flicking, spastic twitching in the hind limbs, along with noticeable swelling and redness of the dura mater post-SCI.

### 2.8 Validation of hub genes via real-time PCR

After the model was successfully established, 10 rats from each group were sacrificed at 2 and 5 days post-operation. All rats were euthanized by cervical dislocation under deep anesthesia with 2% sodium pentobarbital (100mg/kg), followed by transcardial perfusion normal saline (4 °C). The lateral thigh muscles of rats was immediately harvested and stored at -80 °C. Total RNA was extracted using TRIzol reagent (Invitrogen), followed by reverse transcription to synthesize cDNA. PCR amplification was performed, and electrophoresis was used for product detection and data analysis. The PCR conditions were as follows: pre-denaturation at 95 °C for 30 s, denaturation at 94 °C for 1 min, annealing at 56 °C for 1 min, and extension at 72 °C for 90 s, with a total of 35 cycles, followed by a final extension at 72 °C for 10 min. In this study, the internationally commonly used 2^-ΔΔCT^ method was employed to calculate the relative mRNA expression levels.

### 2.9 Statistical analysis

Data analysis was conducted using SPSS 26.0 statistical software(IBM Corporation, Armonk, NY, USA). The Shapiro-Wilk test was used to assess normality. For data that followed a normal distribution, measurements are presented as mean ± SD. To control the family-wise error rate (FWER) arising from multiple comparisons between time points, we applied the Benjamini-Hochberg false discovery rate (FDR) correction. Adjusted p-values were calculated using the GraphPad Prism (version 9.5.0), with a significance threshold set at FDR <0.05. Group differences were analyzed by one-way ANOVA followed by Tukey’s *post hoc* test for pairwise comparisons. Figures were created using Adobe Photoshop CS6 and GraphPad Prism 9.5.0. Statistical significance was set at P<0.05.

## 3 Results

### 3.1 Normalization of data and analysis of differential gene expression

**Figure 1:**
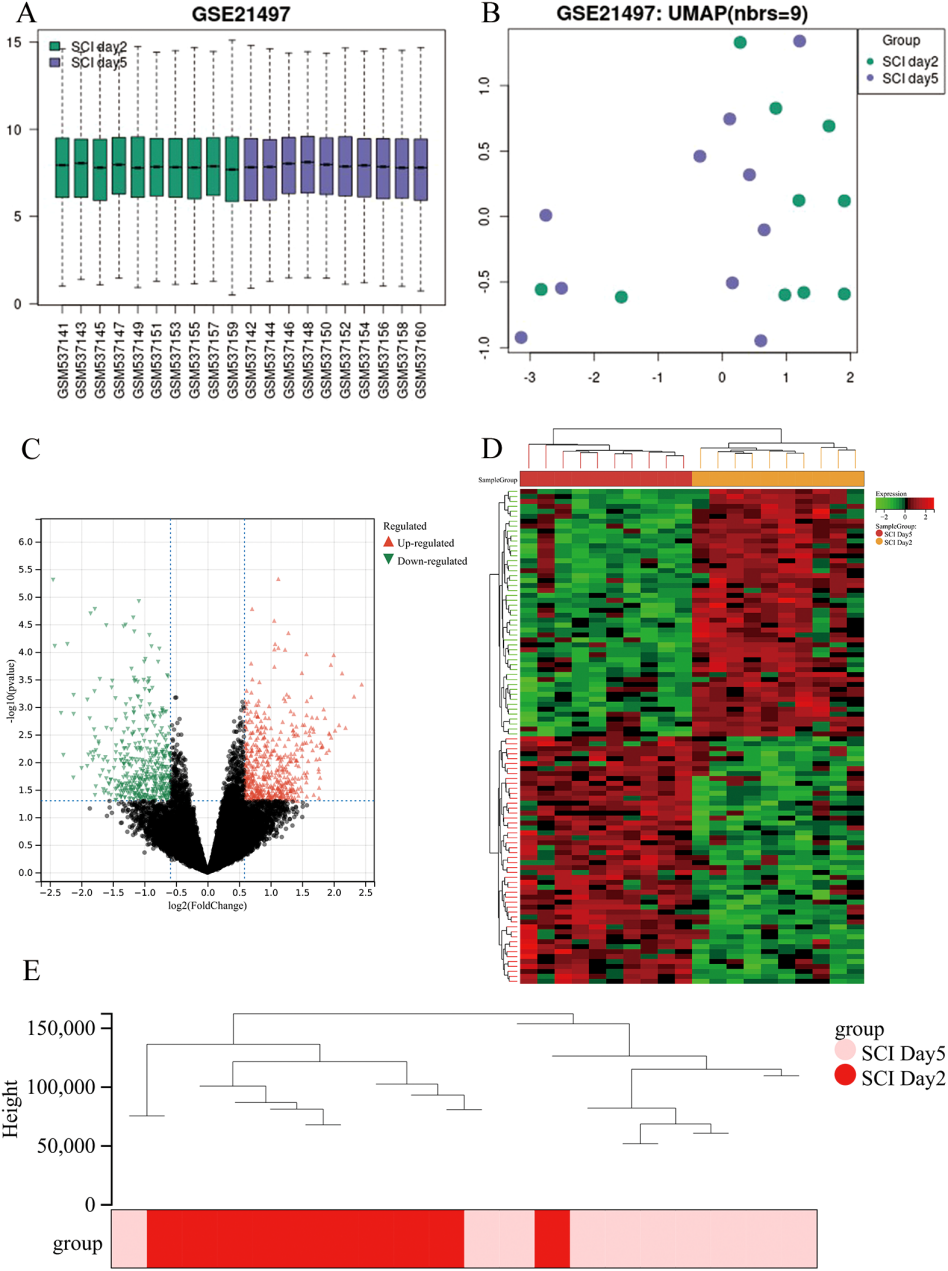
**(A)** Box plots of normalized differential gene expression show that the black lines representing the boxes are generally at similar levels, indicating comparability between the two groups of patient data with minimal deviation between them. **(B)** Uniform Manifold Approximation and Projection (UMAP) algorithm analysis was conducted on all included samples, revealing distinct clustering within both groups and significant differences between the two groups. **(C)** Volcano plot (pink triangles represent upregulated genes, blue-green triangles represent downregulated genes, black dots represent genes with no statistical significance). **(D)** Heatmap (representing differentially expressed proteins between the two groups of patients, where red indicates high expression levels and green indicates low expression levels). **(E)** Perform clustering of differentially expressed genes, where each line represents a gene, and similar genes are clustered into branches.

GSE21497 was downloaded from the GEO database and analyzed using the online tool GEO2R. In the box plot, the black lines are nearly at the same level, indicating minimal deviation between the two (**Figure 1A**). The analysis using the Uniform Manifold Approximation and Projection (UMAP) algorithm reveals distinct clustering between the SCI day 2 group and the SCI day 5 group, indicating significant differences between the two groups (**Figure 1B**). After filtering with *P<*0.05 and |logFC|*>*1.5, a total of 1007 differentially expressed genes (DEGs) were identified on the 5th day post-SCI, including 533 upregulated genes and 474 downregulated genes. Volcano plots and heatmaps were generated using the online Sangerbox 3.0 tool (**Figures 1C, D**).

### 3.2 Data preparation and sample clustering

The data matrix consists of a total of 20 samples, with 1007 DEGs. Further processing was performed using the WGCNA package in R software to conduct sample clustering. The clustering tree was examined to identify and filter out any outliers or anomalies. Based on the visual results of the sample dendrogram, the hierarchical clustering of the samples was obtained (**Figure 1E**).

### 3.3 Selecting the optimal soft threshold

**Figure 2:**
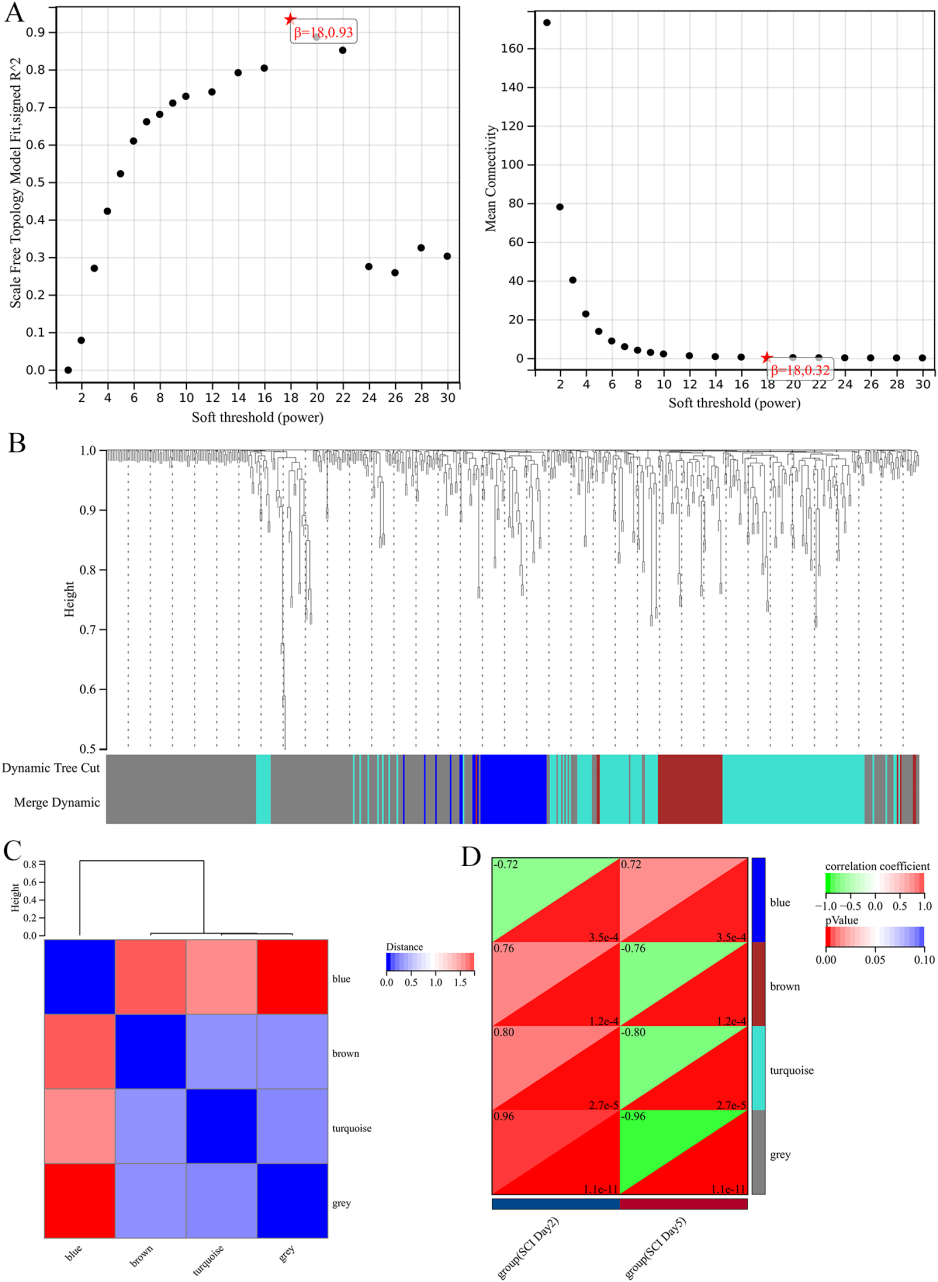
**(A)** Estimation plot of soft threshold for scale-free co-expression networks. **(B)** Dendrogram of hierarchical clustering for all differentially expressed genes. **(C)** Hierarchical clustering dendrogram of module eigengenes. **(D)** Heatmap showing the correlation of each module with the two groups.

After completing the sample clustering observations, the optimal soft threshold (soft thresholding or power) was determined to ensure the constructed network conforms to a scale-free topology. A scatter plot was generated for power values, and the results (**Figure 2A**) showed a clear trend throughout the range from 1, but the trend became less apparent after 18. Therefore, 18 was selected as the optimal power value (with a Power R-squared greater than 0.8). The mean connectivity plot (**Figure 2A**) depicts the network connectivity under different soft threshold values, and the relationship between average connectivity and power values was examined. The connectivity stabilizes at a value of 18, indicating its suitability for power selection.

### 3.4 Constructing co-expression network

Based on the optimal soft threshold, a co-expression network was constructed, and genes were divided into different modules. A gene clustering tree (**Figure 2B**) can be plotted, where the upper part represents the hierarchical clustering dendrogram of genes, and the lower part represents gene modules. Correspondence between the upper and lower parts indicates that genes with closer distances (clustered into the same branch) are assigned to the same module. Each color represents a module, with the grey module considered as a collection of genes that cannot be assigned to any module.

### 3.5 Module correlation clustering and analysis of phenotype-gene correlation

Explore the correlation between various modules, calculate module eigengenes, and plot a network diagram of module eigengenes. Darker colors indicate higher correlation coefficients (**Figure 2C**). Based on the above results, a heatmap of phenotype-gene correlation was plotted(**Figure 2D**). DEGs were categorized into four modules: blue, brown, turquoise, and grey. The color blocks on the right represent modules, while the color bar in the top right corner represents the range of correlations. Darker colors in the heatmap indicate higher correlation, with red indicating positive correlation and green indicating negative correlation. The numbers in each cell represent correlation and significance. Based on the absolute value of correlation, the most relevant modules were selected. The results indicate that the turquoise module (0.80, *p<*0.0001) is the most negatively and positively correlated module with SCI. Therefore, the turquoise module, which includes 161 DEGs, is considered the key module associated with SCI. The grey module contains all genes that were not involved in clustering and is therefore considered an invalid module, not suitable for further analysis.

### 3.6 Functional enrichment analysis

**Figure 3:**
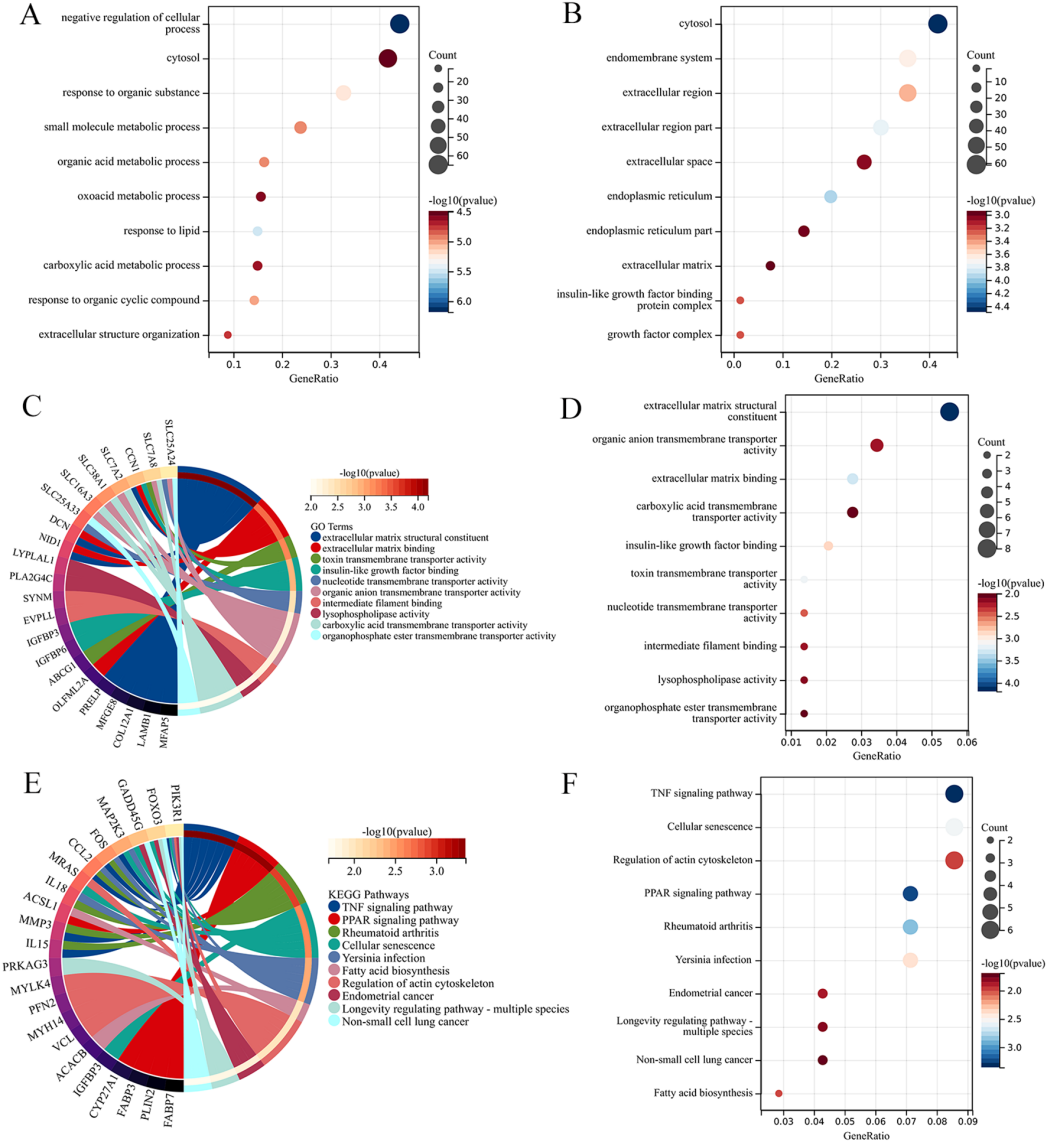
**(A)** The bubble plot to the BP enrichment analysis results for DEGs. **(B)** The bubble plot to the CC enrichment analysis results for DEGs. **(C)** The MF enrichment analysis results for DEGs(Circos plot). **(D)** The MF enrichment analysis results for DEGs (Bubble plot). **(E)** The KEGG enrichment analysis results for DEGs (Circos plot). **(F)** The KEGG enrichment analysis results for DEGs (Bubble plot).

The 161 DEGs in the turquoise module were subjected to GO and KEGG pathway analysis using the DAVID tool. The GO analysis revealed enrichment of the DEGs in 147 biological processes (BP), 146 cellular components (CC), and 145 molecular functions (MF) within the turquoise module. In the BP analysis, the DEGs are significantly enriched in terms such as negative regulation of cellular process, cytosol and response to organic substance(**Figure 3A**). In the CC analysis, the DEGs are significantly enriched in terms such as cytosol, endomembrane system and extracellular region (**Figure 3B**). In the MF analysis, the DEGs are significantly enriched in terms such as extracellular matrix structural, organic anion transmembrane transporter activity constituent and extracellular matrix binding (**Figures 3C, D**). KEGG analysis indicates the involvement of the turquoise module in various signaling pathways including the TNF signaling pathway, cellular senescence and regulation of actin cytoskeleton (**Figures 3E, F**).

### 3.7 Gene set enrichment analysis

**Figure 4:**
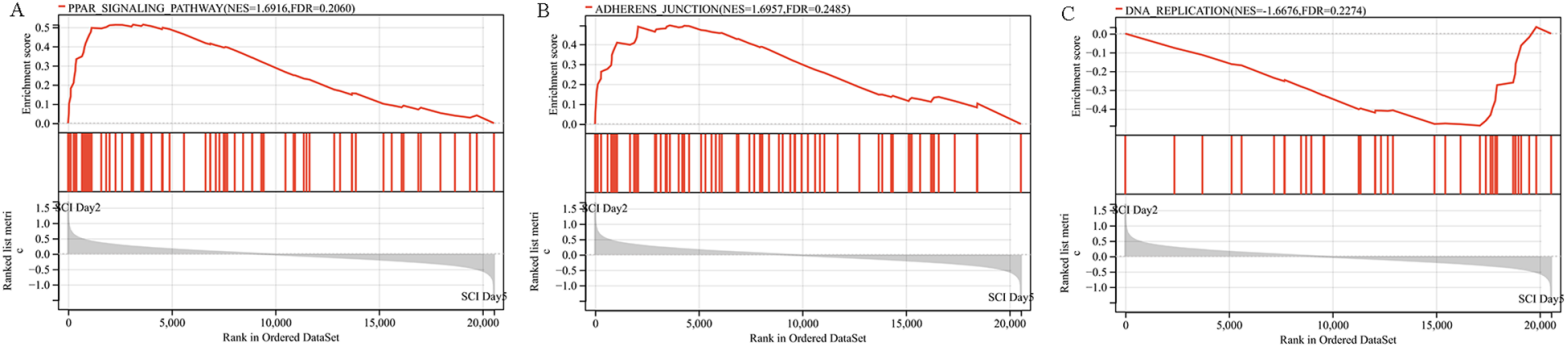
Gene set enrichment analysis plot **(A)** Enrichment plot for the PPARs signaling pathway. **(B)** Enrichment plot for the Adherens Junction signaling pathway. **(C)** Enrichment plot for the DNA Replication signaling pathway.

The Gene Set Enrichment Analysis (GSEA) examined whether there were changes in the distribution of pathway gene sets among different groups of SCI patients. The larger the |NES| value, the smaller the FDR value, indicating higher reliability of the analysis results. The results indicate significant enrichment and upregulation of genes associated with the peroxisome proliferators-activated receptors (PPARs) signaling pathway (NES = 1.6916, FDR = 0.2060) and the adherens junction signaling pathway (NES = 1.6957, FDR = 0.2485) in both groups (**Figures 4A, B**). Conversely, genes related to the DNA replication signaling pathway (NES = -1.6676, FDR = 0.2274) show significant enrichment and downregulation in both groups (**Figure 4C**).

### 3.8 PPI network construction and selection and analysis on hub genes

**Figure 5:**
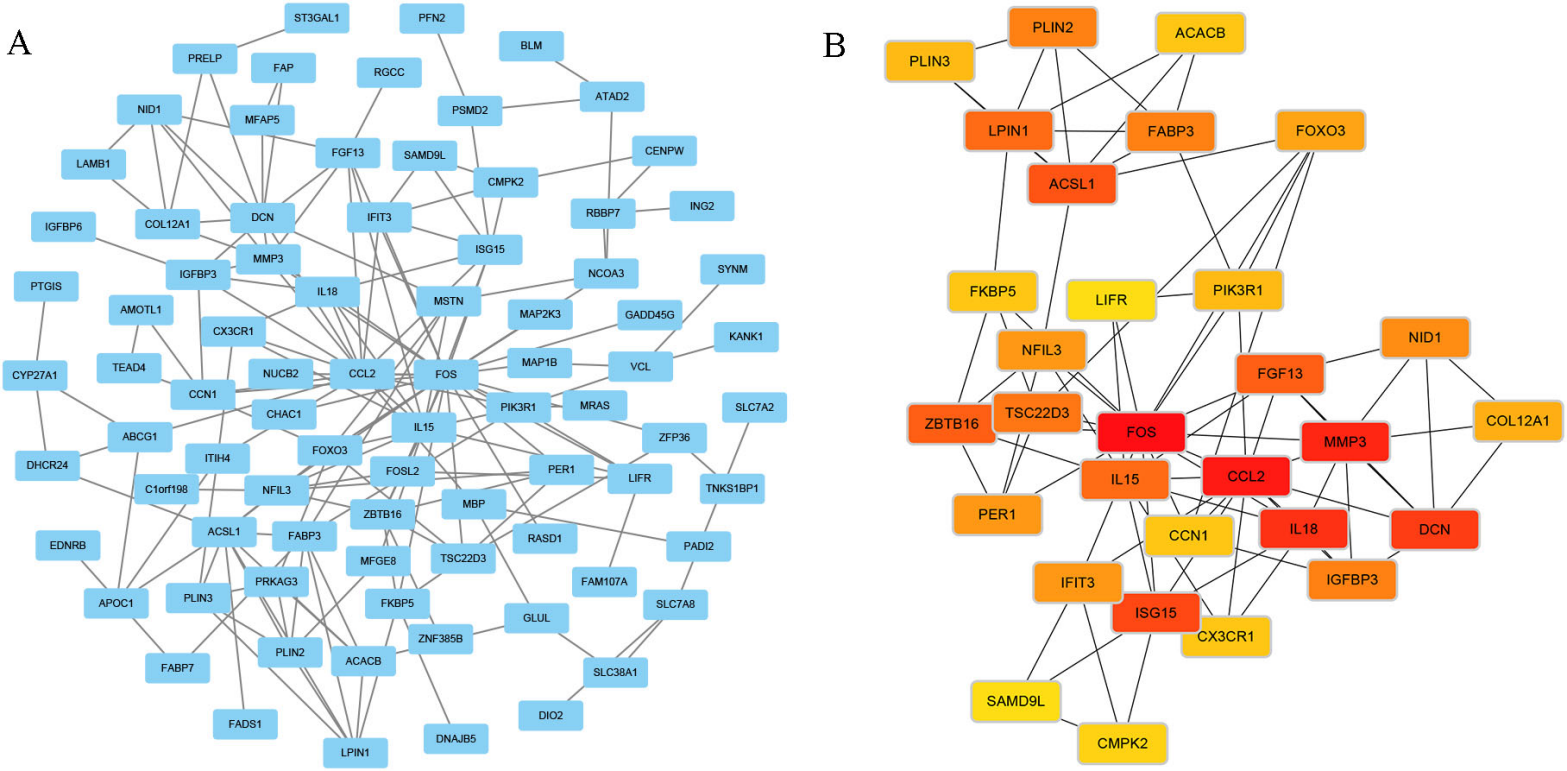
**(A)** Construct a Protein-protein interaction (PPI) network using the Differentially Expressed Genes (DEGs) enriched in the turquoise module, and generate a PPI network diagram. **(B)** Screen the top 30 hub genes and create a network diagram.

**Figure 6:**
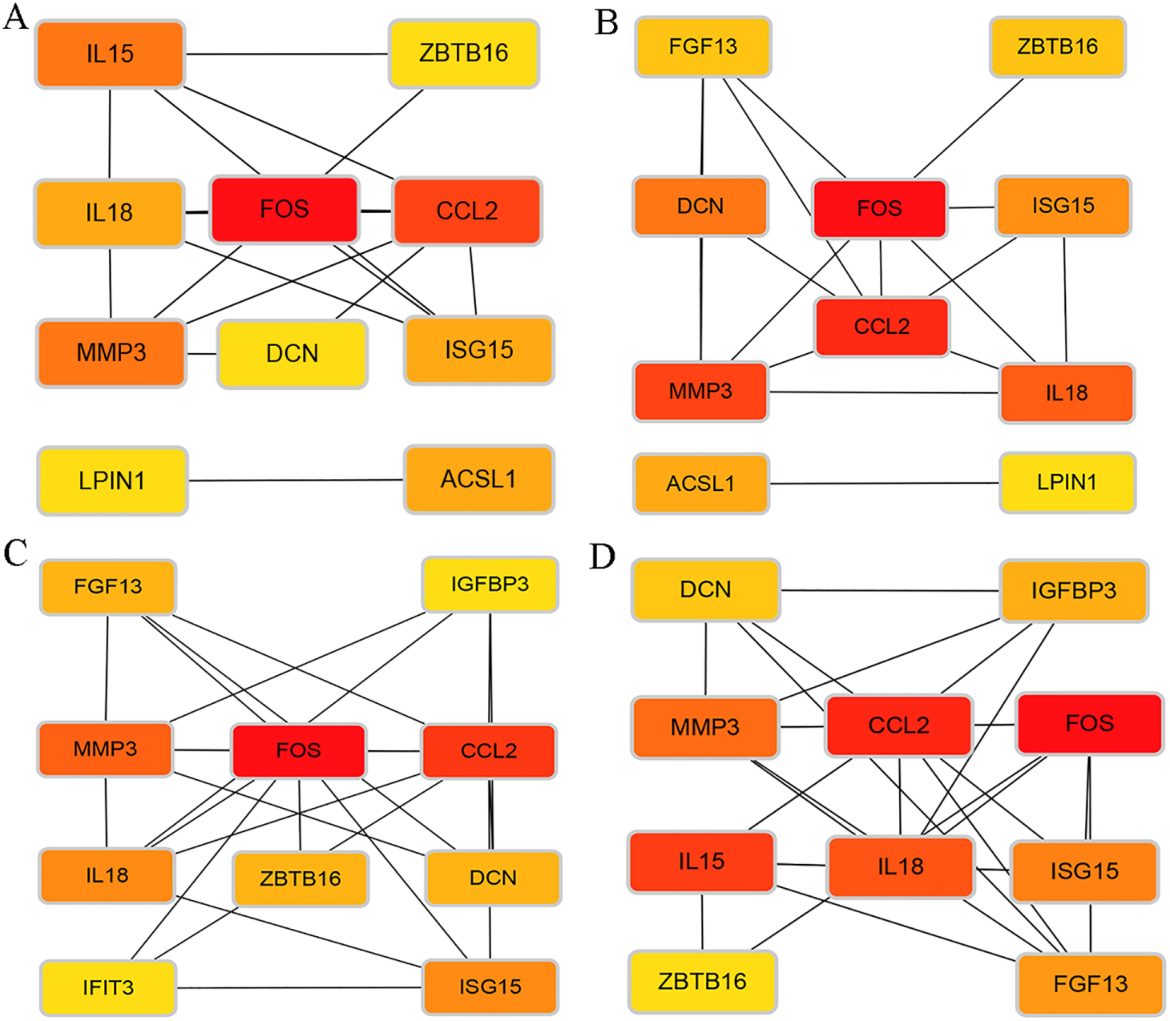
Network diagram of the top 10 critical genes identified by the four algorithms. **(A)** Degree algorithm: evaluates the global connectivity of nodes. **(B)** Maximum Neighborhood Component (MCC) algorithm: focuses on core regulatory nodes. **(C)** Maximum Neighborhood Component (MNC) algorithm: emphasizes the importance within the module. **(D)** Edge Percolated Component (EPC) algorithm: identifies key nodes.

**Figure 7:**
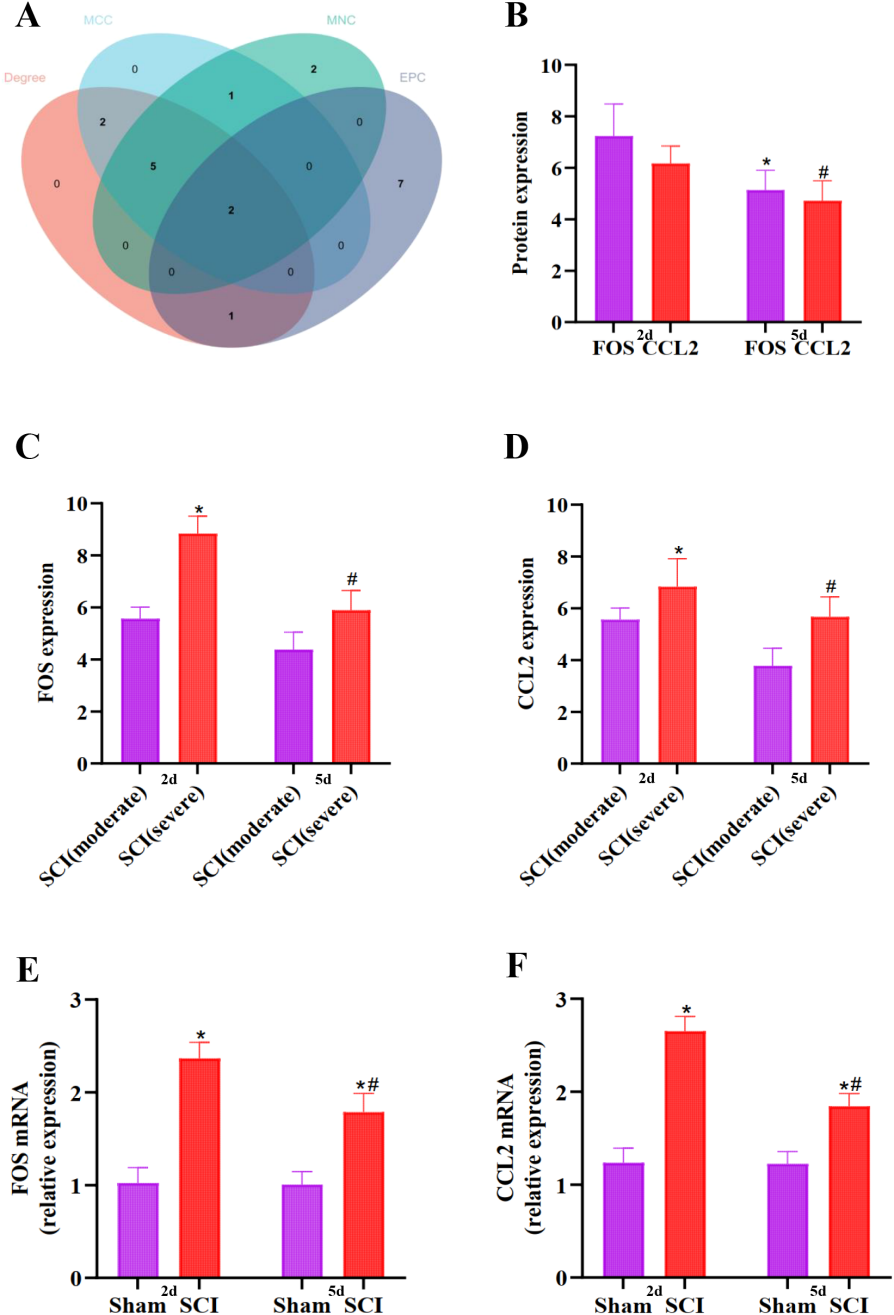
**(A)**: Venn diagram depicting the overlapping hub genes from different algorithms. **(B)**: The expression changes of FOS and CCL2 at different time points were compared, *^#^P<0.05 vs 2 days after SCI. **(C, D)**: Comparison of the expression levels of FOS and CCL2 in the lower limb muscle tissues of patients with SCI of different degrees, *P<0.05 vs SCI (moderate), at 2 days, *^#^P*<0.05 vs SCI (moderate), at 5 days. **(E, F)**: Validation of FOS mRNA and CCL2 mRNA expression in SCI rats via qRT-PCR, *P<0.05 vs Sham group. *^#^P*<0.05 vs SCI group.

Using the STRING database (https://cn.string-db.org), a PPI network was constructed for the enriched DEGs in the turquoise module, with a threshold set to 0.4. The results were exported for further analysis. The file containing node and edge information was imported into Cytoscape using the appropriate plugin, resulting in the PPI network diagram (**Figure 5A**). Subsequently, the top 30 hub genes were selected (**Figure 5B**). Utilizing the CytoHubba plugin in Cytoscape, hub genes were identified using four different calculation methods: Edge Percolated Component (EPC), Maximum Clique Centrality (MCC), Maximum Neighborhood Component (MNC), and Degree. This process aided in identifying the top 10 critical genes (**Figures 6A–D**). The top 10 gene sets obtained from the four different algorithms were input into Sangerbox 3.0 software to determine the number of intersections in the datasets (**Figure 7A**). Finally, FOS and CCL2 were identified as the hub genes for our screening.

Based on the data of 10 samples from the gene expression profile GSE21497 database in this study, it was found that there were differences in the expressions of FOS and CCL2 at 2 days and 5 days after the injury. Specifically, compared with the situation at 2 days after the injury, the protein expressions of FOS and CCL2 both decreased at 5 days after the injury (**Figure 7B**). We analyzed the expression levels of FOS and CCL2 in the lower limb muscle tissues of 6 patients with tetraplegia (severe SCI) and 4 patients with paraplegia (moderate SCI). The results showed that at 2 days, the expression levels of FOS and CCL2 in the lower limb muscle tissues of the 6 tetraplegic patients were higher. By the 5th day, although the expression levels of FOS and CCL2 in their lower limb muscle tissues decreased to some extent, they still remained at a relatively high level (**Figures 7C, D**). This indicates that the higher the expression levels of FOS and CCL2, the more severe the muscle atrophy in patients.

### 3.9 Validation of hub genes

To further validate the reliability of WGCNA results, PCR was used to reconfirm two Hub genes. The primers for gene expression were designed as follows:

FOS: forward 5’-AACAGATCCGAGCAGCTTCTA-3’, reverse5’-TTTTGAGCTTCAACCGGCATC-3’
CCL2: forward 5’-TCTTGCTGCACGTCCTTTATT-3’,
reverse 5’-GCATCCAGTTTTTGTATGTGCC-3’.

The expression of FOS and CCL2 genes in the SCI model was analyzed via PCR. The results of our PCR experiments suggest that the expressions of FOS mRNA and CCL2 mRNA peaked at 2 days after SCI, and then slowly decreased at 5 days after SCI, but still remained at a relatively high level. (**Figures 7E, F**) (*P ≤* 0.05).

## 4 Discussion

The secondary neuropathic changes following SCI are the primary cause of muscle atrophy, which not only impacts the care management and daily activities of SCI patients but also increases the risk of secondary complications (such as osteoporosis, diabetes, and cardiovascular diseases) (25, 26). Therefore, preventing muscle atrophy is crucial for maintaining metabolic health and normal daily activities after SCI. The pathogenesis of muscle atrophy following SCI involves various factors, including signal transduction, immune response, electrical conduction, stimulation, and metabolism (27). SCI causes loss of central regulation function in peripheral nerves below the damaged segment, resulting in sensory, motor, and autonomic dysfunction, muscle paralysis, and decreased muscle loading (28). Impairment of the neuromuscular system and reduced integrity of the musculoskeletal system are critical features of SCI and significant barriers to motor function recovery (29). However, the etiology and precise underlying mechanisms of muscle atrophy after SCI remain poorly understood. The aim of our study is to identify relevant genes, biological processes, and pathway pathways that may play crucial roles in the development of muscle atrophy after SCI, thereby providing theoretical insights into the pathogenesis and therapeutic targets of muscle atrophy following SCI.

In this study, we downloaded the gene dataset GSE21497. After data normalization and standardization, we used the online Sangerbox 3.0 software for computation, resulting in 1007 differentially expressed genes (DEGs) on day 5 after SCI, including 533 upregulated genes and 474 downregulated genes. WGCNA analysis was performed on the data matrix containing 20 samples and 1007 genes with expression differences. WGCNA can identify sets of genes with similar expression patterns, elucidate the relationships between gene sets and sample phenotypes, construct regulatory networks among genes within gene sets, and identify key regulatory genes (30). All 1007 DEGs obtained through the “limma” algorithm were clustered into four modules. It was found that the 161 genes in the turquoise module were most closely related to SCI.

GO enrichment analysis involves examining whether genes in a gene set are enriched at different levels to understand their functions in biological processes (BP), cellular components (CC), and molecular functions (MF) (31). The results of this study show that DEGs are significantly enriched in biological processes such as “negative regulation of cellular process, cytosol and response to organic substance”. DEGs are also significantly enriched in cellular components including “cytosol, endomembrane system and extracellular region”, as well as molecular functions such as “extracellular matrix structural, organic anion transmembrane transporter activity constituent and extracellular matrix binding”. KEGG enrichment analysis is used to analyze the enrichment of gene pathways and functions in a given gene set (32). KEGG analysis reveals that the turquoise module is involved in various signaling pathways such as the “TNF signaling pathway, cellular senescence and regulation of actin cytoskeleton”.

GSEA is used to evaluate the distribution trend of genes from a predefined gene set within a ranked gene expression dataset associated with phenotypes, thereby determining their contribution to the phenotype (33). We utilized the GSEA method to analyze the results, which indicated that following SCI, genes associated with the peroxisome proliferators-activated receptors (PPARs) signaling pathway and adherens junction signaling pathway were significantly increased in both groups, with upregulated expression, while genes related to the DNA replication signaling pathway were significantly decreased in both groups, with downregulated expression. PPARs pharmacological activation can be considered as a multifaceted therapeutic target, exerting anti-inflammatory, antioxidant, antiexcitotoxic, and energy-generating effects in some neurologic and inflammation-related diseases (34). The Adherens junction signaling pathway also demonstrates significant enrichment in comprehensive proteomic and phosphoproteomic analyses following SCI (35), reducing the loss of tight junction and adherens junction proteins in human brain microvascular endothelial cells (HBMECs). This reduction can attenuate blood-spinal cord barrier (BSCB) permeability and degradation of tight junction molecules (such as P120, β-catenin, Occludin, and Claudin5), ultimately improving functional recovery in a traumatic rat model (36). Studies have shown that enhancement of the peroxisome proliferator-activated receptor-γ coactivator-1α (PGC-1α) mitochondrial biogenesis pathway upregulates nuclear respiratory factors (NRF-1,2) and mitochondrial transcription factor A (Tfam), stimulating mitochondrial biogenesis (37). This leads to increased mitochondrial DNA replication and gene transcription, reducing oxidative damage and providing protective effects against atrophy-related skeletal muscle degeneration.

The PPI network illustrates the relationships among proteins and even genes within a given gene set that play a protective role against atrophy-related skeletal muscle degeneration (38). It is one of the commonly used methods for identifying core genes. Cytoscape (https://cytoscape.org/) is an excellent software for graphical visualization and analysis of networks and can be equipped with various functional plugins, making it widely used in PPI analysis (39). Based on the PPI network and the CytoHubba plugin with four different algorithms, the top 10 key genes were identified. These top 10 genes from each algorithm were input into the online Sangerbox 3.0 software for analysis, resulting in a Venn diagram showing FBJ Osteosarcoma Oncogene (FOS) and Chemokine C-C motif ligand 2 (CCL2) as the hub genes we selected. Moreover, the analysis of the data from the gene expression profile GSE21497 shows that the higher the expression levels of FOS and CCL2 are, the more severe the muscle atrophy of the patients will be. It was found that there were differences in the expressions of FOS and CCL2 at 2 days and 5 days after the injury. The results of our PCR experiments suggest that the expressions of FOS mRNA and CCL2 mRNA peaked at 2 days after SCI, and then slowly decreased at 5 days after SCI, but still remained at a relatively high level. Therefore, this dynamic change indicates that inhibiting the expression of CCL2 in the early stage of SCI and upregulating FOS in the chronic stage (such as local injection of FOS overexpression vectors) may be effective methods to alleviate muscle atrophy after SCI.

FOS belongs to a class of genes known as immediate early genes (IEGs). It encodes a nuclear phosphoprotein that serves as a transcription factor (40). FOS plays a significant role in regulating cell growth, division, proliferation, differentiation, and even programmed cell death (41). As the protein product of the *c-fos* gene, under normal conditions, *c-fos* expression is minimal within cells. But, to some extent, the number of FOS-immunoreactive neurons correlates positively with the intensity of the stimulus received, making FOS a marker of injurious stimuli (42). Research has shown that SCI significantly affects *c-fos* expression in the paraventricular nucleus (PVN) and supraoptic nucleus (SON) of the hypothalamus in rats. Following SCI at 1, 6, 12, and 24 hours, there is a significant increase in *c-fos* expression in the PVN, which persists for more than 24 hours. At 12 hours post-SCI, there is a significant increase in *c-fos* expression in the SON, reaching its peak at 24 hours post-SCI (43). As an immediate early gene, FOS can regulate downstream signaling pathways to influence the proliferation, differentiation, and apoptosis of muscle cells, thus participating in the molecular regulatory network of muscle atrophy after SCI (44).

Chemokines are small-molecule peptides secreted by immune or non-immune cells, induced by inflammatory cytokines, growth factors, and pathological stimuli. They play an important role in inflammatory diseases, wound healing, spinal cord injury, and other processes (45). Chemokine C-C motif ligand 2 (CCL2), also known as monocyte chemoattractant protein-1 (MCP-1), is a detrimental factor released after neural injury, primarily involved in the local inflammatory response following SCI (46). After SCI, chronic pain, circulatory disturbances, and mechanical compression lead to changes in neurons of the dorsal root ganglion (DRG). After peripheral nerve or ganglion injury, CCL2 expression is upregulated in DRG neurons (47). Research has shown that CCL2 plays a crucial role in recruiting macrophages during the early stages of muscle injury. Mice lacking CCL2 exhibit incomplete recovery of gastrocnemius muscle mass, muscle fiber cross-sectional area, and gastrocnemius muscle contraction characteristics during disuse atrophy recovery. Additionally, mice lacking CCL2 demonstrate reduced turnover of skeletal muscle collagen and significantly decreased recruitment of macrophages to the gastrocnemius muscle, resulting in poor recovery of muscle size and function as well as abnormal collagen remodeling (48). In muscle atrophy after SCI, C-C motif chemokine ligand 2 (CCL2) can recruit immune cells such as macrophages, exacerbate local inflammatory responses, thereby affecting the balance between metabolism and apoptosis of muscle cells, and promoting the progression of muscle atrophy (49).

We verified the differential expression of FOS and CCL2 in the SCI animal model through PCR, confirming the reliability of the bioinformatics analysis. The potential role of FOS: As an activator protein-1(AP-1) transcription factor, it may promote the activity of the ubiquitin-proteasome system by activating the Atrogin-1/MuRF1 pathway, thus facilitating the degradation of muscle fibers (50). The potential role of CCL2: Recruiting macrophages through the CCR2 receptor, inducing the release of pro-inflammatory factors (such as TNF-α and IL-6), and accelerating the apoptosis of muscle cells (51). These analyses link the changes in gene expression with known pathological mechanisms, pointing the way for subsequent functional studies. In terms of drug research and development, the already marketed JNK inhibitor (such as CC-930) can indirectly inhibit the activity of FOS by blocking the formation of the AP-1 complex (51). The CCL2 antagonist (Bindarit, a CCL2 synthesis inhibitor) can reduce the infiltration density of macrophages and improve neuromotor function (52). In terms of clinical translation, the serum CCL2 level can be used as a biomarker to predict the effective rate of Bindarit treatment and guide patient stratification. Clinical trials of the synergistic use of FOS inhibitors and rehabilitation training can also be carried out.

Based on the existing research, the pro-inflammatory and pro-atrophic mechanisms of FOS and CCL2 make them ideal targets for the treatment of muscle atrophy following SCI. For example, the small molecule inhibitors T-5224 (targeting FOS/AP-1) and RS504393 (targeting CCL2/CCR2) have demonstrated anti-inflammatory and anti-fibrotic effects in other disease models (53, 54). Future studies could verify whether these drugs alleviate muscle atrophy in the SCI model by inhibiting the MuRF1/MAFbx pathway or regulating macrophage polarization. Additionally, combining gene silencing techniques or existing neurotrophic therapies may produce synergistic effects. In conclusion, as hub genes, FOS and CCL2, through the closed-loop path of “network screening-mechanism analysis-drug verification-clinical transformation”, provide powerful targets for the development of precise drugs against muscle atrophy.

## 5 Limitations

Our study has several limitations. Firstly, the GSE21497 dataset used in this study is from the GEO database. Although it provides valuable information on the gene expression of muscle atrophy after SCI, there are still some limitations such as sample heterogeneity, and the lack of clinical information. Secondly, the samples included in this study are concentrated within 5 days after injury, which may not fully reflect the molecular characteristics of muscle atrophy in the chronic phase. Therefore, there may be a bias in temporal dynamics during the sample selection process. Finally, we have not directly verified the quantitative relationship between the expression of FOS and CCL2 and the severity of muscle atrophy in patients. In the later stage, we will continue to conduct clinical research related to this project.

## 6 Conclusions

In summary, we utilized WGCNA to analyze the expression profile data from GSE21497, identifying two hub genes, FOS and CCL2, in muscle atrophy following SCI. FOS and CCL2 may be involved in the molecular pathophysiology of muscle atrophy after SCI, serving as potential targets for diagnosis or treatment of SCI-related muscle atrophy.

## Data Availability

The datasets presented in this study can be found in online repositories. The names of the repository/repositories and accession number(s) can be found in the article/supplementary material.
